# Inhibition of CK2 Diminishes Fibrotic Scar Formation and Improves Outcomes After Ischemic Stroke via Reducing BRD4 Phosphorylation

**DOI:** 10.1007/s11064-024-04112-0

**Published:** 2024-02-21

**Authors:** Xuemei Li, Qinghuan Yang, Peiran Jiang, Jun Wen, Yue Chen, Jiagui Huang, Mingfen Tian, Jiangxia Ren, Qin Yang

**Affiliations:** 1 Department of Neurology, The First Affiliated Hospital of Chongqing Medical University, 1 Youyi Road, Yuzhong District, Chongqing, 400016 China; 2 Department of Neurology, The Second People’s Hospital of Chongqing Banan District, Chongqing, China

**Keywords:** CK2, p-BRD4, Fibroblast, Fibrotic scar formation, Cerebral ischemia

## Abstract

Fibrotic scars play important roles in tissue reconstruction and functional recovery in the late stage of nervous system injury. However, the mechanisms underlying fibrotic scar formation and regulation remain unclear. Casein kinase II (CK2) is a protein kinase that regulates a variety of cellular functions through the phosphorylation of proteins, including bromodomain-containing protein 4 (BRD4). CK2 and BRD4 participate in fibrosis formation in a variety of tissues. However, whether CK2 affects fibrotic scar formation remains unclear, as do the mechanisms of signal regulation after cerebral ischemic injury. In this study, we assessed whether CK2 could modulate fibrotic scar formation after cerebral ischemic injury through BRD4. Primary meningeal fibroblasts were isolated from neonatal rats and treated with transforming growth factor-β1 (TGF-β1), SB431542 (a TGF-β1 receptor kinase inhibitor) or TBB (a highly potent CK2 inhibitor). Adult SD rats were intraperitoneally injected with TBB to inhibit CK2 after MCAO/R. We found that CK2 expression was increased in vitro in the TGF-β1-induced fibrosis model and in vivo in the MCAO/R injury model. The TGF-β1 receptor kinase inhibitor SB431542 decreased CK2 expression in fibroblasts. The CK2 inhibitor TBB reduced the increases in proliferation, migration and activation of fibroblasts caused by TGF-β1 in vitro, and it inhibited fibrotic scar formation, ameliorated histopathological damage, protected Nissl bodies, decreased infarct volume and alleviated neurological deficits after MCAO/R injury in vivo. Furthermore, CK2 inhibition decreased BRD4 phosphorylation both in vitro and in vivo. The findings of the present study suggested that CK2 may control BRD4 phosphorylation to regulate fibrotic scar formation, to affecting outcomes after ischemic stroke.

## Introduction

After nervous system injury, the inflammatory response is activated, new tissue forms and tissue remodeling occurs [1]. Fibrotic scar formation is involved in tissue repair and reconstruction in the late stage of brain injury. Scars in the CNS include glial and fibrotic scars [1, 2]. Glial scars, located around the injury site, play a role in preventing neuronal degeneration, inhibiting axonal regeneration, and reconstructing the blood‒brain barrier [3, 4]. Fibrotic scars, located at the center of the injury, are composed of a variety of cells with different characteristics and origins (such as the meninges and peripheral blood vessels) [5–7]. These cells migrate to the injury site, transform into myofibroblasts, and secrete extracellular matrix components such as collagen, laminin and fibronectin to form fibrotic scars [8–11]. At present, the role of fibrotic scars in tissue repair and remodeling after CNS injury is unclear. A few studies have shown that fibrotic scars prevent synaptogenesis and aggravate neurological deficits during the remodeling period after brain injury [6, 9, 12]. However, recent studies have shown that complete prevention of fibrotic scarring can lead to cavity formation and hinder functional recovery after spinal injury [13]. Therefore, it is important to elucidate the mechanism of fibrotic scar formation after cerebral ischemic injury to improve patient prognosis.

Casein kinase II (CK2), a messenger-independent serine/threonine protein kinase, is widely present in the cytoplasm and nucleus of eukaryotic cells [14]. CK2 comprises two catalytic subunits (α and/or α′) and two regulatory subunits (β), and it phosphorylates a variety of substrates and participates in cell growth, proliferation, apoptosis, carcinogenesis and other processes [15, 16]. 4,5,6,7-Tetrabromobenzotriazole (TBB) is an efficient and selective ATP competitive inhibitor of CK2, which has a smaller hydrophobic pocket adjacent to the ATP/GTP binding site than do the majority of other protein kinases [17]. TBB enters cells readily and appears not to be cytotoxic in the short term, which makes it useful for experiments [18]. Many studies have shown that CK2 regulates the occurrence and development of fibrosis, inflammation, metabolic diseases, nervous system diseases and tumors [19–23]. CK2 phosphorylates a variety of substrates and regulates fibrosis in peripheral tissues, such as the liver, lung, kidney, and skin [14, 24–26]. Moreover, CK2 is widely expressed in the central nervous system, such as in neurons, microglia, and astrocytes, and regulates neuronal survival, glial cell regeneration, synaptic regeneration and remodeling after stroke [27–30]. However, it remains unclear whether and how CK2 affects fibrotic scar formation after cerebral ischemia injury.

Bromodomain-containing protein 4 (BRD4), a member of the bromodomain and extraterminal domain (BET) family, binds to transcription factors and acetylated histones to recruit multiple transcriptional regulators to control inflammation, chromatin assembly, oxidative stress injury, and cell proliferation [31–33]. BRD4 is highly clustered at gene enhancer sites of genes and regulates fibrosis in a variety of tissues and organs [34–39]. CK2 and protein phosphatase type 2A (PP2A) regulate BRD4 phosphorylation and dephosphorylation, respectively. There are two CK2 phosphorylation sites (NPS and CPS) in the BRD4 domain [40, 41]. CK2 binds to phosphorylation sites to produce phosphorylated BRD4, which binds to acetylated chromatin and specific transcription factors to regulate site-specific gene transcription [40–43]. Our previous results showed that BRD4 participates in fibrotic scar formation after brain injury [44]. However, whether CK2 regulates fibrotic scar formation after cerebral ischemia by phosphorylating BRD4 has not been determined.

Here, we hypothesized that CK2 inhibition could reduce the activation of fibroblasts induced by TGF-β1 and attenuate fibrosis after cerebral ischemic injury by reducing BRD4 phosphorylation. We found that in vitro, CK2 expression was upregulated in a TGF-β1-induced meningeal fibroblast fibrosis model, and in vivo, CK2 expression was upregulated in a middle cerebral artery occlusion/reperfusion (MCAO/R) injury model. Treatment with SB431542, a TGF-β1 receptor kinase inhibitor, decreased CK2 expression in fibroblasts. The highly potent CK2 inhibitor TBB decreased the proliferation, migration and activation of fibroblasts caused by TGF-β1 in vitro, inhibited fibrotic scarring, ameliorated histopathological damage, reduced Nissl body damage and improved neurological function after MCAO/R injury in vivo. Moreover, CK2 inhibition decreased BRD4 phosphorylation both in vitro and in vivo. This study is the first to indicate that CK2 may control BRD4 phosphorylation to regulate fibrotic scar formation and affect outcomes after MCAO/R injury.

## Materials and Methods

### Reagents

4,5,6,7-Tetrabromobenzotriazole (TBB) (HY-14394), transforming growth factor-β1 (TGF-β1) (HY-P70543) and SB431542 (HY-10431) were obtained from MedChemExpress (USA). Dimethyl sulfoxide (DMSO; D2650) was obtained from Sigma (USA). A rabbit polyclonal anti-BRD4 primary antibody (DF2905) was obtained from Affinity Company (USA). A rabbit polyclonal anti-phospho-BRD4 (Ser492/Ser494) primary antibody (ABE1453) was obtained from Merck Millipore Company (USA). A polyclonal rabbit anti-CK2α primary antibody (GTX107897) was obtained from GeneTex International Corporation (USA). Alexa Fluor 594-conjugated goat anti-mouse (550042) and Alexa Fluor 488-conjugated goat anti-rabbit (550037) secondary antibodies were obtained from Zen Bioscience (Cheng Du, China). Polyclonal mouse anti-α-SMA (α-SMA; 55135-1-AP), rabbit anti-GAPDH (10494-1-AP), polyclonal mouse anti-FN (66042-1-Ig), HRP-conjugated AffiniPure goat anti-mouse (SA00001-1) and goat anti-rabbit IgG (SA00001-2) antibodies were obtained from Proteintech (Wuhan, China). Monoclonal rabbit anti-CK2β (ab76025) and a Picrosirius red staining kit (ab150681) were obtained from Abcam (USA). The hematoxylin–eosin (HE) staining kit (G1120), 2,3,5-triphenyltetrazolium chloride (TTC) solution (G3005), Nissl staining kit (methyl violet method) (G1432) and 4% paraformaldehyde (P1110) were obtained from Beijing Solarbio Science & Technology (Beijing, China). A 5-ethynyl-2ʹ-deoxyuridine (EdU) cell proliferation kit with Alexa Fluor 488 (C0071S) and a BCA protein assay kit (P0012S) were obtained from Beyotime Biotechnology Company (Shanghai, China). DMEM/F12 (C11330500BT) was obtained from Gibco (USA). The PCR primers used were obtained from Sangon Biotech (Shanghai, China). Fetal bovine serum (FBS; P30-2602) was obtained from PAN-Biotech (Germany).

### Experimental Animals

Neonatal (1–2 days old) and adult male (weight, 200–300 g) Sprague–Dawley (SD) rats were purchased from the Department of Animal Experiments, Chongqing Medical University (Chongqing, China). All adult rats were housed in a temperature-controlled room at a humidity of 40–70% and a temperature of 24–26 °C on a 12 h light/dark cycle and provided free access to food and water. This study was approved by the Animal Experimental Committee of Chongqing Medical University (Ethics Committee Code: 2021-560) and complied with relevant laws such as the IMPROVE and ARRIVE guidelines [45, 46]. The number of rats in each group was predetermined based on previously published studies. Furthermore, researchers blinded to the experimental setup evaluated the outcomes.

### Cell Culture

Meningeal fibroblasts from neonatal SD rats were isolated and cultured as previously described by Li et al. [44]. Briefly, the meninges were separated and digested into single-cell suspensions with IV collagenase (1 mg/ml). Fibroblasts were cultured in DMEM/F-12 medium supplemented with 1% penicillin/streptomycin and 10% fetal bovine serum and passaged after they reached confluence. Second- to fourth-generation fibroblasts were used for subsequent studies.

### MCAO/R Model

Focal cerebral ischemia was induced by middle cerebral artery occlusion/reperfusion (MCAO/R) in adult SD rats via the insertion of filaments into the artery to occlude blood for 120 min, as described by Longa et al. [47]. Rats that underwent the same surgical procedure but without the insertion of fibrous filaments into the lumen were used for comparison. Rats that developed subarachnoid hemorrhage, died, or had no neurological impairment after surgery were not included in the study.

### Drug Treatment

The profibrotic factor TGF-β1 (10 ng/ml) [24, 48–50], the TGF-β1 receptor kinase inhibitor SB431542 (20 µM) [51, 52], and the CK2 inhibitor TBB (5 µmol/l) [24, 53] are commonly used for in vitro studies of fibrosis in a variety of tissues. In our previous research, BRD4 protein expression in primary meningeal fibroblasts was significantly upregulated by 10 ng/ml TGF-β1 and downregulated by 20 µM SB431542 in vitro [44, 52]. TBB (2.5 mg/kg/d and 10 mg/kg/d) administered by intraperitoneal injection inhibited fibrosis in a dose-dependent manner in vivo [14, 24].

Therefore cells were incubated in vitro with TGF-β1 (10 ng/ml), SB431542 (20 µM), dimethyl sulfoxide (DMSO) or TBB (5 µmol/l) for 3 days to evaluate the expression of CK2α and CK2β and the activation of fibroblasts. The solvent SB431542, DMSO or TBB was added to the medium 1 h before TGF-β1 treatment.

In vivo, TBB (5 mg/kg/d) was injected intraperitoneally in volumes of 50 μL or less per rat once a day starting on the day after ischemia‒reperfusion. CK2 expression and its effects on fibrotic scar formation were evaluated after MCAO/R injury.

### Neurological Deficit Score

Neurological deficit scores were evaluated at 1, 7 and 14 days after MCAO/R via the modified neurological severity score (mNSS) [54], Bederson score [55], and Longa score [47] by an independent investigator. The higher the score is, the more severe the neurological deficit.

The mNSS was used for comprehensive assessment of motor, sensory, reflex, and balance functions. A higher mNSS (0 points, normal; 18 points, loss of consciousness or death) indicates more severe neurological deficits.

The Bederson score was used to measure the forelimb flexion of rats suspended 10 cm, as follows: forelimb extension (0 points); forelimb flexion (1 point); decreased resistance to lateral push (2 points); spontaneous rotation (3 points); circling behavior, decreased activity (4 points).

The Longa score was used to evaluate motor function as follows: normal (0 points); limited left limb movement (1 point); paralysis, walking in circles or rear-end collision (2 points); paralysis, falls or rolls, unable to stand (3 points); and no activity or disorder of consciousness (4 points).

### RT‒PCR Analysis

The mRNA levels of CK2α, CK2α’ and CK2β in TGF-β1-treated meningeal fibroblasts were analyzed at 72 h via RT‒PCR. Cultured meningeal fibroblasts were washed and collected. Total RNA was extracted from fibroblasts with TRIzol. Table 1 shows the CK2α, CK2α’, CK2β and β-actin primer sequences. Quantitative PCR (qPCR) was carried out on a CFX96 Touch Real-Time PCR Detection System (Bio-Rad, Hercules, USA). The 2^−ΔΔCt^ method, in which β-actin was used for normalization, was used to estimate the amount of target mRNA in the samples. All the samples were analyzed in triplicate.

**Table 1:** Primers for RT‒PCR

Gene name	Sense (5ʹ–3ʹ)	Antisense (3ʹ–5ʹ)
Csnk2α1	CTGGACAAGCTGCTGCGATACG	CTGCCATGCCAGCCGAACTC
Csnk2α2	CTTCTTGACAAGCTCCTGCGGTAC	AGGCTGGGACTGCTCCTTCAC
Csnk2β	CTTCGGCACTGGTTTCCCTCAC	TTGAAGTTGCTGGCGGCTTGG
β-actin	GGCACCCAGCACAATGAAG	CCGATCCACACGGAGTACTTG

### EdU Assay

The proliferation of meningeal fibroblasts was examined with a 5-ethynyl-2’-deoxyuridine (EdU) cell proliferation kit. First, fibroblasts were seeded in 96-well plates and treated as described above. Second, EdU solution was added to each well, and the cells were incubated for 2 h at 37 °C. Then, the cells were fixed in 4% paraformaldehyde for 15 min and subsequently washed with PBS. Afterward, the cells were stained with Click solution for 30 min at room temperature and DAPI for 15 min at 37 °C. The cells were analyzed with a fluorescence microscope (Olympus, Japan). The cell proliferation rate was detected by the ratio of EdU-positive cells to total cells. The experiments were repeated 3 times.

### Scratch Wound Assay

Cell migration was measured with a scratch assay. The meningeal fibroblast monolayer was scratched and incubated with different media for 48 h. Three random fields of the scratch at different time points (0 and 48 h) were observed with a microscope, and the percentage of the wound-healed areas was analyzed by ImageJ software. The experiments were repeated 3 times.

### TTC Staining

The infarct volumes in the MCAO/R model rats were examined via TTC staining. Rats were euthanized at 7 days after reperfusion (*n* = 3). The brains were chilled at − 20 °C for 20 min to slightly harden the tissue. Six 2 mm coronal sections were cut with a brain matrix (model number 68700; RWD Life Science, Shenzheng, China) on ice and stained with 1.0% TTC solution at 37 °C for 30 min. The infarcts generated by MCAO/R were observed in the striatum and the dorsolateral cortex. The striatum was found to be more sensitive to ischemia than the cerebral cortex [56]. The infarct area was white, whereas the normal tissue was stained red. The stained brain sections were imaged with a digital camera. The infarct volumes were quantified by ImageJ software analysis and calculated by the formula: Infarct volume (%) = (contralateral volume—ipsilateral noninfarct volume)/contralateral volume × 100 [57].

### HE Staining

Rat brain samples were embedded into paraffin blocks and serially sectioned into 4 µm coronal sections. Three nonconsecutive brain slices per rat were randomly selected for each subsequent staining.

A hematoxylin–eosin (HE) staining kit was used to assess the histological structure of the brain. Briefly, brain sections were sequentially stained with hematoxylin solution for 5 min and differentiated with ethanol hydrochloride solution for 3 s and eosin solution for 3 min at room temperature. Three randomly selected regions of interest in the ischemic core of the striatum and the cerebral cortex in each brain slice were photographed under a microscope with blinding at a magnification of ×200. Brain damage after MCAO/R was evaluated by observing the ischemic core structures of the striatum and the cerebral cortex, such as the nucleus (purple‒blue), cytoplasm and extracellular matrix (pink). All the experiments were repeated 3 times.

### Nissl Staining

A Nissl staining kit (with the methyl violet method) was used to assess the Nissl bodies in cerebral ischemia in rats. The brain sections were treated with methyl violet staining solution for 20 min and washed with Nissl differentiation medium for approximately 8 s. Three randomly selected regions of interest in the ischemic core of the striatum and the cerebral cortex in each brain slice were photographed under a microscope at a magnification of ×200. Dark purple‒blue indicates Nissl bodies. Normal neurons had large cell bodies with abundant cytoplasm and obvious Nissl bodies. Pyknosis or blurring of Nissl bodies suggested neuronal damage. The number of Nissl bodies was calculated as the area ratio of the Nissl bodies to the image. The results of each section were averaged and statistically analyzed with ImageJ software. All the experiments were repeated 3 times.

### Sirius Red Staining

The brain sections were incubated with Picrosirius red solution for 60 min at room temperature and washed with an acetic acid solution. Three randomly selected regions of interest in the ischemic core of the striatum and the cerebral cortex in each brain slice were photographed under a microscope at a magnification of ×200. Collagen fibers were stained red with Sirius red solution. The area of collagen fibers was calculated as the percentage of the image area occupied by collagen fibers. The results for each section were averaged and statistically analyzed with ImageJ software. All the experiments were repeated 3 times.

### Immunocytochemistry and Immunofluorescence

Fibroblasts were fixed with paraformaldehyde (4%). The brain sections were microwaved to retrieve the antigen. Afterward, the brain sections or cells were blocked with goat serum (10%) and treated with polyclonal rabbit anti-CK2α antibody (1:100), polyclonal mouse anti-α-SMA antibody (1:100), monoclonal rabbit anti-CK2β antibody (1:100), or polyclonal mouse anti-FN antibody (1:100). For negative controls, PBS was used instead of antibody. After being washed with PBS, the sections or cells were incubated with Alexa Fluor 594-conjugated goat anti-mouse IgG (1:100) and/or Alexa Fluor 488-conjugated goat anti-rabbit IgG (1:100) for 1 h at 37 °C. DAPI staining solution was used to stain the cell nuclei. Finally, the cells were observed and photographed under an A1 + R laser confocal microscope (Nikon, Tokyo, Japan). Three random fields of the central infarct area were selected from each slice. All immunocytochemistry and immunofluorescence assays were repeated three times.

### Western Blot Analysis

Protein was extracted from cells or brain tissues from the ischemic core according to the manufacturer’s instructions. The protein concentrations of the extracts were assessed by a BCA protein assay kit. Equal amounts of protein from different groups were separated via SDS–PAGE and subsequently transferred to PVDF membranes. After blocking with nonfat milk (5%), the PVDF membranes were treated with rabbit anti-p-BRD4 antibody (1:2000), rabbit anti-BRD4 antibody (1:2000), rabbit anti-CK2α antibody (1:1000), rabbit anti-CK2β antibody (1:1000), mouse anti-α-SMA antibody (1:2000), rabbit anti-GAPDH antibody (1:10,000) or mouse anti-FN antibody (1:4000). Then, the membranes were incubated with HRP-conjugated AffiniPure goat anti-mouse IgG (1:5000) or goat anti-rabbit IgG (1:5000) secondary antibodies for 1 h. A Bio-Rad instrument was used to quantify the protein bands. Fusion software was used for semiquantitative analysis of protein expression. The gray ratio of the target protein to GAPDH was used to normalize the expression level of the target protein. All the samples were analyzed in triplicate.

### Statistical Analysis

SPSS 20.0 for Windows was used to perform the statistical analyses. Quantitative data are presented as the means ± standard deviations (SDs). The results were analyzed with Bonferroni’s or Tukey’s post hoc test after one-way or two-way analysis of variance (ANOVA). Single comparisons were analyzed by Student’s t test. Neurological deficit scores are expressed as medians and ranges and were analyzed with the Kruskal‒Wallis test followed by Dunn’s post hoc test. *P* < 0.05 was considered to indicate statistical significance.

## Results

### CK2α and CK2β Expression is Increased in a TGF-β1-Induced Fibrosis Model In Vitro and an MCAO/R-Induced Fibrotic Scar Formation Model In Vivo

**Fig. 1:**
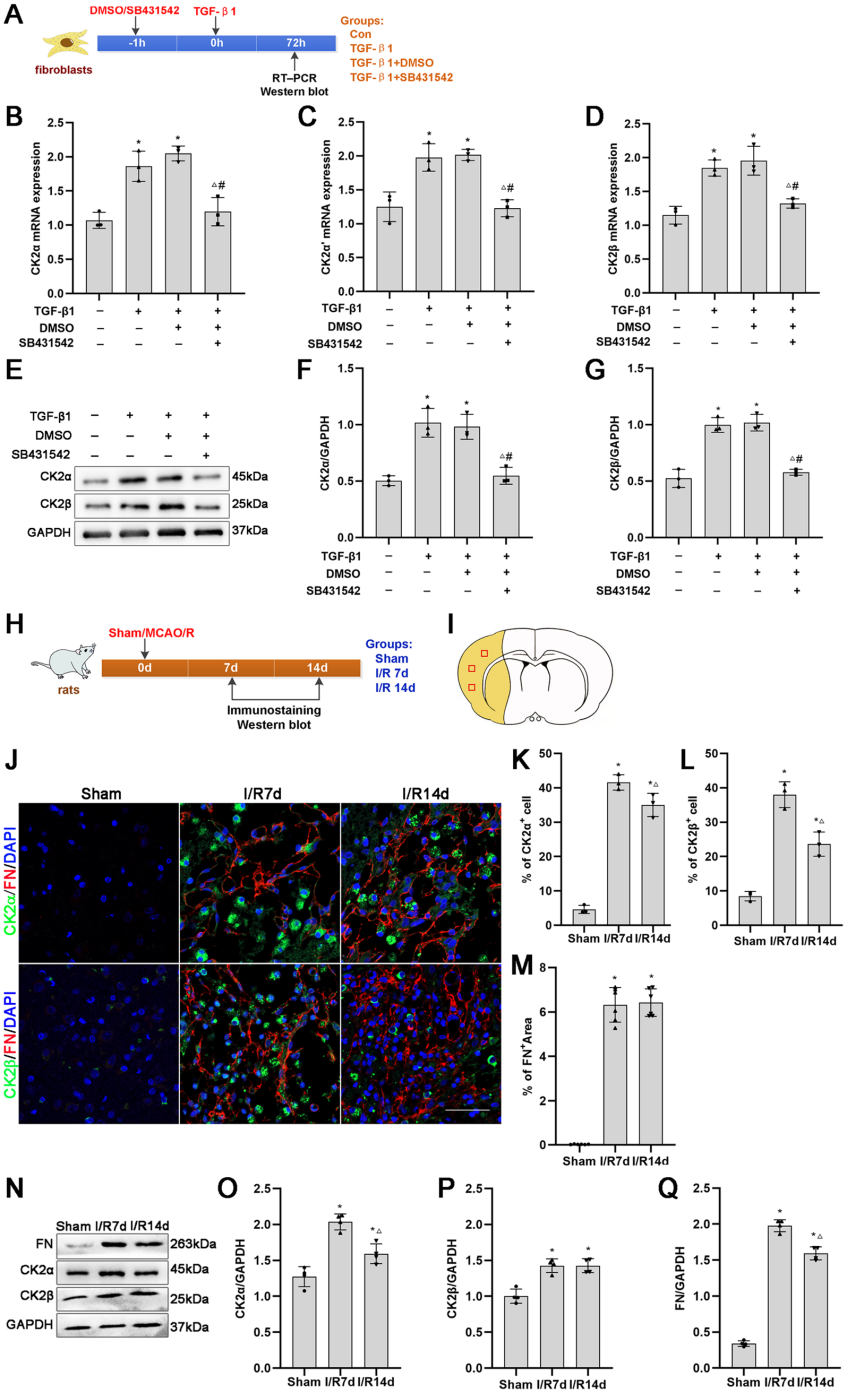
CK2α and CK2β expression was increased in vitro in the TGF-β1-induced fibrosis model and in vivo in the MCAO/R injury model. **A** Timeline of fibroblast treatment. **B**–**D** RT‒PCR analysis of CK2α, CK2α′ and CK2β mRNA expression in each group of fibroblasts treated with TGF-β1 for 72 h (*n* = 3). **E**–**G** Representative protein expression and quantification data for CK2α and CK2β in each group of fibroblasts were obtained by Western blotting (*n* = 3). *P < 0*.*05 vs. the Con group; ^△^*P* < 0*.*05 vs. the TGF-β1 group; ^#^*P* < 0.05 vs. the TGF-β1 + DMSO group. **H** Timeline and group diagram of the rats. **I** Schematic diagram of the coronal plane of the brain. The ischemic core is represented by the yellow area. The observed regions are shown as square boxes. **J** Immunofluorescence staining of FN^+^/CK2α^+^ and FN^+^/CK2β^+^ cells and images of merged signals in the ischemic core after cerebral ischemia (*n* = 3). Red represents tissue immunostained with antibodies against FN, and green represents cells immunostained with antibodies against CK2α or CK2β. Scale bars: 50 μm. **K**–**M** Representative quantitative analysis of FN-positive areas and CK2α-positive and CK2β-positive cells. **N**–**Q** Protein expression and quantification of FN, CK2α and CK2β levels in the ischemic core after cerebral ischemia caused by MCAO/R, as detected by Western blotting (*n* = 3). *P < 0.05 vs. the sham group; ^△^*P* < 0.05 vs. the I/R 7 d group

TGF-β1 is an important contributor to fibrosis. We first investigated whether CK2 expression is altered in a TGF-β1-induced meningeal fibroblast fibrosis model in vitro. RT‒PCR analysis revealed that the expression levels of CK2α, CK2α' and CK2β mRNAs in the TGF-β1 and TGF-β1 + DMSO groups were significantly greater than those in the Con and TGF-β1 + SB431542 groups (Fig. 1B–D). In addition, Western blotting analysis showed that the protein expression levels of CK2α and CK2β in the TGF-β1 and TGF-β1 + DMSO groups were significantly greater than those in the Con and SB431542 groups (Fig. 1E–G). The results suggested that CK2 expression was upregulated by TGF-β1 and downregulated by SB431542 in meningeal fibroblasts, which indicated that CK2 may play a role in TGF-β1-induced meningeal fibroblast fibrosis.

Next, we investigated whether fibrotic scar formation and CK2 expression were affected after MCAO/R injury. Immunofluorescence and Western blotting demonstrated that the expression of CK2α, CK2β and fibronectin (FN; a marker of fibrotic scarring) in the infarct core at 7 and 14 days after MCAO/R was significantly greater than that in the sham group (Fig. 1J–Q). Moreover, the protein expression of CK2α and FN in the I/R7d group was significantly greater than that in the I/R14d group (Fig. 1J–Q). These results indicated that cerebral ischemic injury significantly induced fibrosis and upregulated the expression of CK2α and CK2β in the infarct core at 7 days. Therefore, in the subsequent study, observation was performed 7 days after MCAO/R.

Our study showed that the expression of CK2α and CK2β was upregulated in the TGF-β1-induced fibrosis model and MCAO/R injury model.

### TBB Inhibits the Proliferation, Migration and Activation of Meningeal Fibroblasts In Vitro

The above findings indicate that CK2 expression was upregulated in fibrosis models both in vitro and in vivo. We further investigated whether CK2 inhibition by TBB, a selective ATP-competitive inhibitor of CK2, affects the proliferation, migration and activation of meningeal fibroblasts caused by TGF-β1 in vitro.

**Fig. 2:**
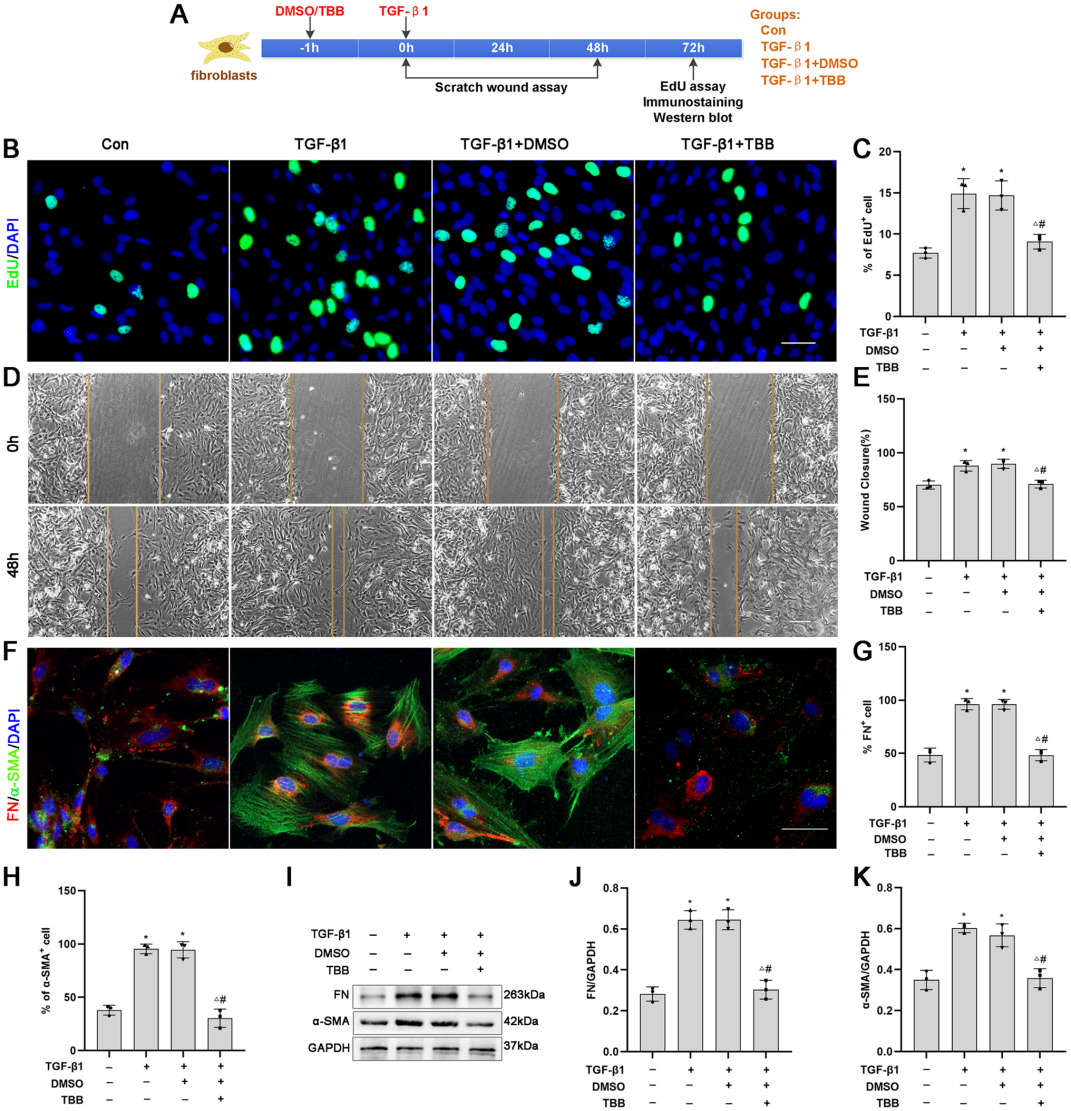
Effects of TBB on the proliferation, migration and activation of fibroblasts induced by TGF-β1 in vitro. **A** Timeline of fibroblast treatment. **B**, **C** Fibroblast proliferation in each group induced by TGF-β1 for 72 h was detected by EdU analysis. Green and blue represent EdU-positive cells and nuclei, respectively (*n* = 3). Scale bars: 50 μm. **D**, **E** The migration of fibroblasts in each group was detected by the scratch wound assay (*n* = 3). Scale bars: 200 μm. **F**–**H** The activation of fibroblasts in each group was detected by immunofluorescence staining with antibodies against α-SMA and FN. Green indicates the α-SMA, and red indicates the FN. (*n* = 3). Scale bars: 50 µm. **I**–**K** Representative protein expression and quantification analysis of α-SMA and FN protein levels in fibroblasts induced by TGF-β1 for 72 h, as detected by Western blotting (*n* = 3). *P < 0.05 vs. the Con group; ^△^*P* < 0.05 vs. the TGF-β1 group; ^#^*P* < 0.05 vs. the TGF-β1 + DMSO group

The proliferation, migration and secretion of extracellular matrix from fibroblasts are involved in fibrous scar formation. EdU and scratch wound assays showed that cell proliferation and migration were significantly upregulated in the TGF-β1 and TGF-β1 + DMSO groups and downregulated in the TGF-β1 + TBB group (Fig. 2B–C, D–E). α-SMA is a marker of fibroblast activation, and FN is an extracellular matrix component secreted by activated fibroblasts. Immunofluorescence analysis demonstrated that α-SMA and FN protein expression was significantly upregulated in the TGF-β1 and TGF-β1 + DMSO groups and downregulated in the TGF-β1 + TBB group compared with the control group (Fig. 2F–H). Western blotting analysis also showed that TGF-β1 up-regulated the expression of the α-SMA and FN proteins, and TBB had the opposite effect (Fig. 2I–K).

These results indicated that CK2 could regulate the proliferation, migration and activation of fibroblasts induced by TGF-β1.

### TBB Inhibits Fibrosis, Ameliorates Damage, Protects Nissl Bodies, and Improves Outcomes After MCAO/R Injury

TBB inhibited TGF-β1-induced fibroblast activation in vitro. We further investigated whether CK2 inhibition by TBB affects fibrotic scar formation and outcomes after MCAO/R injury in vivo.

**Fig. 3:**
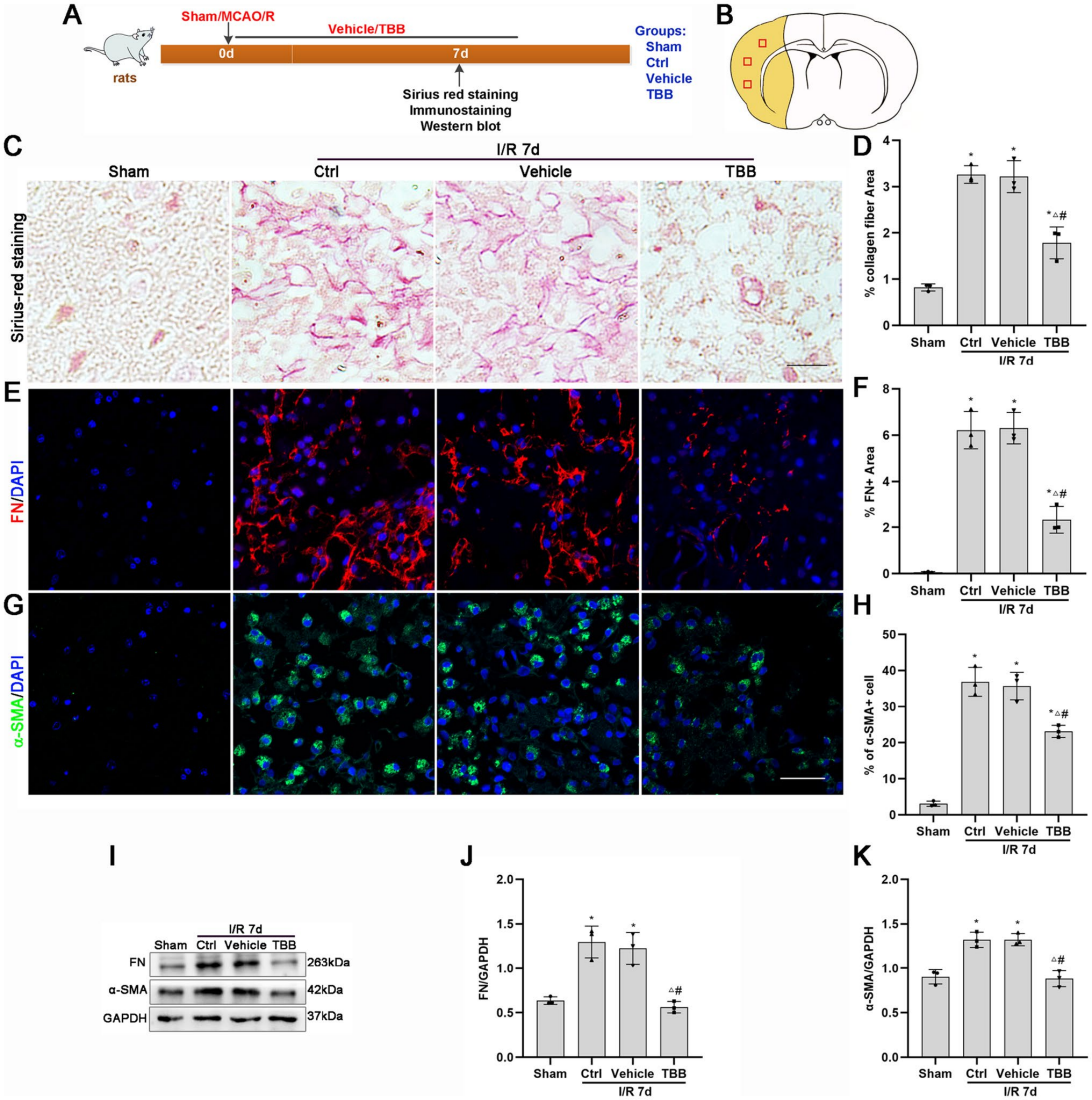
TBB reduces fibrosis after MCAO/R injury. **A** Timeline and group diagram of the rats. **B** Schematic diagram of the coronal plane of the brain. The ischemic core is represented by the yellow area. The regions of interest are shown as square boxes. **C**, **D** Expression and quantitative analysis of collagen fibers with Sirius red staining at 7 days after MCAO/R (*n* = 3). Red represents collagen fibers. Scale bars: 1 µm. **E**–**H** Protein expression levels and quantitative analysis of FN and α-SMA at 7 days after cerebral ischemic injury determined by immunofluorescence staining (*n* = 3). Red represents FN-positive areas. Green represents α-SMA-positive cells. Blue represents nuclei. Scale bars: 40 μm. **I**–**K** Protein expression levels and quantitative analysis of α-SMA and FN in the ischemic core at 7 days after MCAO/R injury determined by Western blotting (*n* = 3). *P < 0.05 vs. the sham group; ^△^*P* < 0.05 vs. the Ctrl group; ^#^*P* < 0.05 vs. the vehicle group

Collagen fibers, FN and α-SMA are markers of fibrotic scar formation. Collagen fibers are fibrous components that consist primarily of collagen and amino acids. Under a light microscope, the collagen fibers were stained red with a yellow background by Sirius red staining. Sirius red staining, immunofluorescence and Western blotting revealed significantly more collagen fibers and significantly higher α-SMA and FN protein expression in the infarct core in the Ctrl, vehicle and TBB groups than in the sham group and lower in the TBB group than in the Ctrl and vehicle groups (Fig. 3C–K). These results suggested that TBB inhibited fibrous scar formation in the ischemic core after MCAO/R injury.

**Fig. 4:**
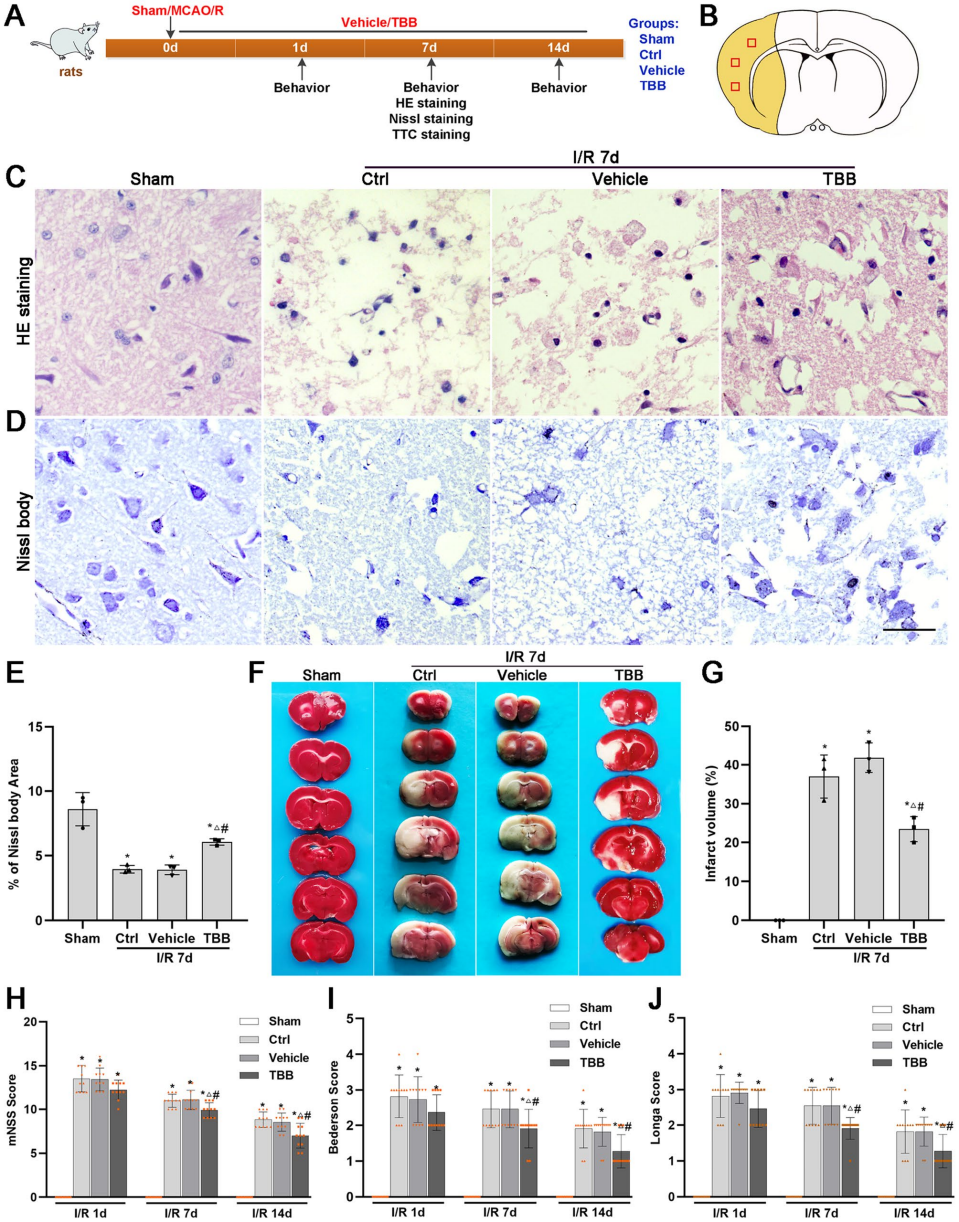
TBB ameliorates histopathological damage, protects Nissl bodies, decreases infarct volume and ameliorates neurological deficits after MCAO/R injury. **A** Timeline and group diagram of the rats. **B** Schematic diagram of the coronal plane of the brain. The ischemic core is represented by the yellow area. The observed regions are shown as square boxes. **C**, **D** Representative histological structure assessed by HE staining and Nissl body (bluish violet) expression assessed by Nissl staining of the ischemic core at 7 days after MCAO/R injury (*n* = 3). Scale bars: 2 µm. **E** Quantitative analysis of Nissl bodies expressed as the area ratio of the Nissl bodies to the image. **F**, **G** Representative images and quantitative analysis of the infarct volume in brain slices after MCAO/R determined by TTC staining (*n* = 3). White indicates infarction, while red indicates normal tissue. Analysis of neurological function according to the mNSS (**H**), Bederson score (**I**), and Longa score (**J**) at 1, 7 and 14 days after MCAO/R injury (*n* = 12). *P < 0.05 vs. the sham group; ^△^*P* < 0.05 vs. the Ctrl group; ^#^*P* < 0.05 vs. the vehicle group

Hematoxylin and eosin (H&E) staining was performed to assess histopathological damage after MCAO/R injury. The results showed that the structure of the striatum and the cerebral cortex in the sham group was normal. In these rats, the neurons were densely and evenly arranged, with a normal structure and morphology, clear cell contours, and clearly visible and intact nucleoli. In contrast, the histological structure of the brain was disrupted after MCAO/R injury, with tissue necrosis, neuronal loss and marked glial cell infiltration in the ischemic core. However, the morphological changes in the TBB group were less severe than those in the Ctrl and vehicle groups (Fig. 4C).

Nissl bodies are basophilic masses and granules composed of rough endoplasmic reticulum and free ribosomes in the cell body or dendrites of neurons and are markers of neurons. The Nissl body staining results showed neurons with large cell bodies, abundant cytoplasm and obvious Nissl bodies in the sham group and neurons with pyknosis or blurred Nissl bodies in the Ctrl, vehicle and TBB groups. Moreover, the number of Nissl bodies in the infarct core was significantly lower in the Ctrl and vehicle groups than in the sham group and greater in the TBB group than in the Ctrl and vehicle groups (Fig. 4D, E).

The infarct volume was evaluated via TTC staining to assess brain injury at 7 days after MCAO/R. As shown in Fig. 4F and G, there was no cerebral infarction in the sham group. The infarct volume was significantly lower in the TBB group than in the Ctrl and vehicle groups. The results showed that TBB significantly decreased the infarct volume after MCAO/R.

Neurological deficits in the rats were evaluated with the mNSS, Bederson score, and Longa score at 1, 7 and 14 days after MCAO/R injury (Fig. 4H–J). There was no neurological deficit in the sham group. The rats in the Ctrl, vehicle and TBB groups exhibited obvious neurological deficits after MCAO/R, which were gradually alleviated over time. There were significantly fewer neurological deficits in the TBB group than in the Ctrl and vehicle groups at 7 and 14 days, but there was no significant difference at 1 day after MCAO/R. The results showed that neurological deficits after stroke were gradually alleviated in a time-dependent manner. TBB significantly improved stroke outcomes, with better outcomes observed at 14 days than at 7 days.

These results showed that the CK2 inhibitor TBB decreased fibrotic scar formation, reduced brain damage and Nissl body loss, and improved neurological outcomes after MCAO/R.

### TBB Inhibits BRD4 Phosphorylation in a TGF-β1-Induced Fibrosis Model In Vitro and an MCAO/R Injury Model In Vivo

The above studies showed that CK2 is involved in the TGF-β1-induced activation of meningeal fibroblasts in vitro and in fibrotic scar formation after MCAO/R injury in vivo. We further investigated whether CK2 regulates fibrosis by affecting BRD4 phosphorylation.

**Fig. 5:**
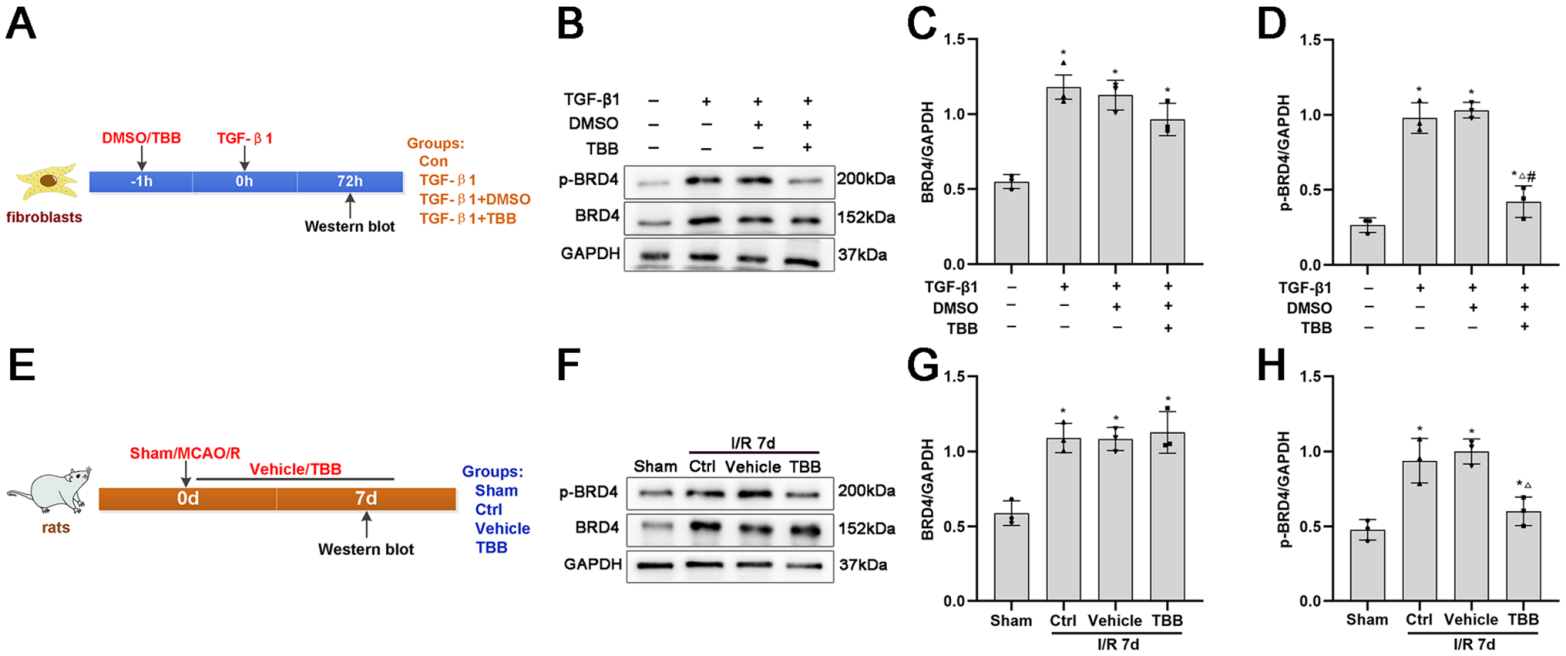
Effects of TBB on BRD4 phosphorylation in vitro and in vivo. **A** Timeline of fibroblast treatment. **B**–**D** Representative protein expression and quantitative analysis of p-BRD4 and BRD4 protein levels in fibroblasts incubated with TGF-β1 and/or TBB for 72 h, determined by Western blotting (*n* = 3). *P < 0.05 vs. the Con group; ^△^*P* < 0.05 vs. the TGF-β1 group; ^#^*P* < 0.05 vs. the TGF-β1 + DMSO group. **E** Timeline and group diagram of the rats. **F**–**H** Representative protein expression and quantitative analysis of p-BRD4 and BRD4 protein levels in the ischemic core at 7 days after cerebral ischemia injury, as assessed by Western blotting (*n* = 3). *P < 0.05 vs. the sham group; ^△^*P* < 0.05 vs. the Ctrl group; ^#^*P* < 0.05 vs. the vehicle group

Western blotting revealed that the protein levels of BRD4 and p-BRD4 were increased in the in vitro TGF-β1-induced fibrosis model and in the in vivo MCAO/R injury model (Fig. 5A–H). The CK2 inhibitor TBB decreased the level of p-BRD4 but not the BRD4 protein in vitro (Fig. 5A–D). Moreover, TBB had a similar effect on the ischemic core after MCAO/R (Fig. 5E–H). These results showed that the CK2 inhibitor TBB reduced BRD4 phosphorylation both in vitro in a TGF-β1-induced fibrosis model and in vivo during MCAO/R injury-induced fibrotic scar formation, which suggested that CK2 regulates fibrosis by phosphorylating BRD4 in vitro and in vivo.

## Discussion

The present study showed that CK2 expression was upregulated in vitro in a TGF-β1-induced meningeal fibroblast fibrosis model and in vivo in a middle cerebral artery occlusion (MCAO)/reperfusion (R) injury model. Treatment with SB431542, a TGF-β1 receptor kinase inhibitor, decreased CK2 expression in fibroblasts. The highly potent CK2 inhibitor TBB decreased the proliferation, migration and activation of fibroblasts caused by TGF-β1 in vitro, inhibited fibrotic scarring, ameliorated histopathological damage, reduced Nissl body damage and improved neurological function after MCAO/R injury in vivo. Moreover, CK2 inhibition decreased BRD4 phosphorylation in vitro and in vivo. This study is the first to indicate that CK2 may control BRD4 phosphorylation to regulate fibrotic scar formation and affect outcomes after MCAO/R injury.

CK2 is a protein kinase that occurs widely in eukaryotic cells and participates in cell growth, proliferation, apoptosis and other processes. CK2 is composed of two catalytic subunits (α and/or αʹ) and regulatory subunits (β). Several studies suggest that the α catalytic subunit has important kinase activity and is the primary functional subunit for disease treatment [14, 58, 59]. The β subunit is likely responsible for the oncogenic potential of CK2. In epithelial–mesenchymal transition (EMT), TGF-β signaling increases CK2 activity by decreasing the protein level of CK2β without affecting the protein level of CK2α [48]. Many studies have shown CK2 participates in liver, lung, skin and kidney fibrosis [24, 26, 60]. In systemic sclerosis fibroblasts, CK2α and CK2β expression was also increased. Here, we found that CK2α and CK2β expression was upregulated during fibrotic scarring after stroke and in a TGF-β1-induced fibrosis model, respectively. These findings suggest CK2 may participate in the regulation of fibrotic scarring after cerebral ischemia, which is consistent with previous findings.

CK2 regulates tissue fibrosis through a variety of signaling pathways, such as the TGF-β1/Smad3, p-NF-κB [25], Wnt/β-catenin [26], IKK/NF-κB [14], and JAK2/STAT3 pathways [24] and non-Smad signaling pathways (Akt and Erk) [61]. CK2 also controls tissue and cellular functions by phosphorylating a variety of substrates. For example, CK2 regulates BRD4 function by binding to phosphate sites (NPS and CPS) in the BRD4 domain [40]. CK2-mediated BRD4 phosphorylation promotes resistance to BET inhibitors in lung adenocarcinoma, while CK2 inhibitors reduce resistance [62]. In drug-resistant triple-negative breast cancer cells, decreased PP2A activity causes hyperphosphorylation of BRD4 [63]. In addition, the CK2 inhibitor TBB or CK2 knockout reduces neuronal immediate early gene expression and inhibits synaptic remodeling and memory generation by controlling BRD4 phosphorylation [64]. Here, our results showed that CK2 regulates fibrous scar formation after MCAO/R injury by affecting BRD4 phosphorylation. Therefore, CK2 may play multiple roles through multiple signaling pathways or through the phosphorylation of multiple substances in different organs.

CK2 occurs widely in neurons, astrocytes and myelin sheaths and participates in a variety of functions in the central nervous system [27, 28]. For example, CK2 inhibited NADPH oxidase-triggered oxidative stress injury and reduced neuronal death 24 h after brain injury [30]. CK2 regulated the activation and migration of microglia induced by ischemia and hypoxia and controlled glial scar formation [65]. CK2 inhibition could promote oligodendrocyte survival, protect axonal structure and axonal mitochondrial function, and improve neurological function after cerebral ischemia [53, 66, 67]. Moreover, CK2 regulates platelet synthesis, activation and thrombosis, and CK2β knockout significantly reduces cerebral infarct volume, alleviates neurological deficits and improves the prognosis in rats with cerebral ischemia [68]. Similarly, our findings showed that CK2 controlled fibroblast activation and fibrosis at 7 days after cerebral infarction. These studies suggest that CK2 plays different or even opposing roles in different tissues.

In addition, the study had several limitations. For example, CK2 is expressed in almost all mammalian cells. We intraperitoneally injected the CK2 inhibitor TBB to detect fibrous scar formation after cerebral infarction in vivo. Whether intraperitoneal TBB acted on other cells, such as platelets, endothelial cells and immune cells, and thereby indirectly affected the repair of cerebral infarction is unknown. Therefore, lateral intraventricular administration of TBB will be used to reduce the effect of TBB on peripheral tissues and cells in future research. In addition, fibrous scar formation after stroke involves a variety of cells, such as meningeal fibroblasts, pericytes and stromal cells. Whether and how these cells interact with each other needs to be further investigated.

## Conclusion

Our present data demonstrate that CK2 may control BRD4 phosphorylation to regulate fibrotic scar formation and affect outcomes after ischemic stroke. Our findings provide important insights into the role of CK2 in fibrotic scarring after cerebral ischemic injury and may open new avenues for stroke treatment. However, we will further investigate how p-BRD4 affects fibroblast activation and fibrotic scarring in vivo and in vitro.

## Data Availability

The data supporting the findings are available from the corresponding author upon reasonable request.

## Abbreviations

BRD4: Bromodomain-containing protein 4
CK2: Casein kinase II
HE: Hematoxylin–eosin
TRKI: TGF-β receptor kinase inhibitor
TGF-β1: Transforming growth factor-β1
PP2A: Protein phosphatase type 2A
SD: Sprague–Dawley
TNBC: Triple-negative breast cancer
MCAO/R: Middle cerebral artery occlusion/reperfusion
NADPH: Nicotinamide adenine dinucleotide phosphate
BET: Bromodomain and extraterminal domain
ICAM-1: Intercellular cell adhesion molecule-1
α-SMA: α-Smooth muscle actin
p-BRD4: Phosphorylated bromodomain-containing protein 4
DMSO: Dimethyl sulfoxide
TBB: 4,5,6,7-Tetrabromobenzotriazole
FN: Fibronectin
EdU: 5-Ethynyl-2ʹ-deoxyuridine
ANOVA: One-way analysis of variance
mNSS: Modified neurological severity score
